# Polish Physicians’ Perspectives on Medical Cannabis Policy and Educational Needs: Results of An Online Survey

**DOI:** 10.3390/jcm10194545

**Published:** 2021-09-30

**Authors:** Martyna Hordowicz, Jerzy Jarosz, Małgorzata Czaplińska, Agnieszka Leonhard, Anna Klimkiewicz

**Affiliations:** 1 Hospice of St. Christopher in Warsaw, 02-781 Warsaw, Poland; malgorzata.czaplinska@fho.org.pl (M.C.); agnieszka.leonhard@fho.org.pl (A.L.); 2 Polish Society of Medical Cannabis and Cannabinoids, 02-781 Warsaw, Poland; jerzy.jarosz@op.pl (J.J.); anna.klimkiewicz@wum.edu.pl (A.K.); 3 Medical Faculty, Medical University of Warsaw, 02-781 Warsaw, Poland

**Keywords:** medical cannabis, Poland, physicians, perspectives, survey, cannabinoids, education

## Abstract

(1) Background: In November 2017, medical cannabis was legalized in Poland. Until now, there have been no studies conducted to examine the perspectives of Polish physicians about their preferences regarding medical cannabis legal status and educational needs. (2) Methods: The survey was a self-developed online questionnaire with 57 participants. Participation was voluntary. The link was shared through a personal network of medical doctors, regional medical chambers, and with doctors attending palliative care courses organized by our research group. Results: Between June and October 2020, 173 HCPs from Poland completed the survey. More than half of the study participants never received any education on medical cannabis (60.1%); 71.1% declared their knowledge was insufficient to counsel patients about medical cannabis use. The majority claimed that they would like to be able to answer patient questions (92.4%); 93.1% declared a need to create clear guidelines for using cannabinoids in clinical practice. Furthermore, 71.7% believed that medicines containing cannabinoids and 52.0% that herbal cannabis should be reimbursed (3). Conclusion: Most medical doctors do not feel prepared for patient counseling. They could benefit from targeted educational interventions. We have also identified physicians’ preferences that might inspire the stakeholders involved who are critical for shaping policies regarding cannabis-based therapeutics.

## 1. Introduction

On the 1 November 2017, the legal status of herbal cannabis in Poland changed. It became legal as a pharmaceutical raw material for preparing prescription drugs material under the Act of Counteracting Drug Addiction in Poland [1]. Physicians may prescribe cannabis under the same conditions as other controlled substances. Several strains are available in Poland, with varying THC: CBD (tetrahydrocannabinol: cannabidiol) ratio and terpenoid and flavonoid profile. Sativex, oromucosal spray with THC, i.e., a CBD ratio close to 1:1, is also registered in Poland for symptom improvement in auld patients with moderate to severe spasticity due to MS (multiple sclerosis) [2,3]. The Ministry of Health (MoH) did not grant the refund to any medicine containing cannabinoids, which means that the patients pay the total, relatively high cost of treatment. There were only a few exceptions where patients were granted financing from MoH through a compassionate use program.

Current Polish regulations classify THC as a substance with a high probability of abuse and low therapeutic value (II-P group). Herbal cannabis, other than fiber-type cannabis (with THC content ≥0.2%), is classified under the I-N category, defined as a substance with high abuse potential, which may have therapeutic applications [4,5]. There are no defined limits on the quantity of possessed cannabis for treatment purposes. However, it should be no higher than the amount required for 90 days of treatment, which is calculated based on dosing on the prescription of THC, with no upper limit; this regulation does not apply to other cannabinoids, such as CBD [6].

The cultivation of cannabis, including culture by patients’ medical needs, is not allowed on Polish territory. Therefore, the whole market relies on deliveries of cannabis from foreign countries by marketing authorization holders (MAHs) registered in Poland, originating from Canada or Germany. The sole exception is the culture of cannabis for research purposes, but such permission is granted only in exceptional situations by the Chief Sanitary Inspectorate [6].

The Polish the Agency for Health Technology Assessment and Tariffication (HTA&T) has repeatedly recommended against reimbursement of herbal cannabis on numerous conditions: chemotherapy-induced nausea and vomiting; multiple sclerosis; chronic pain; epilepsy, including treatment-resistant epilepsy; Dravet syndrome; Jacobsen syndrome, and glaucoma due to Sturge–Weber Syndrome [7,8,9,10]. All the HTA&T statements point out that there is no evidence demonstrating the effectiveness of specific, CBD-rich cannabis strains in refractory epilepsy. However, they admit that there is evidence of the benefits of this active compound from clinical trials of other medicines in both Dravet and Sturge–Weber Syndromes [7,8]. A similar narrative the HTA agency used to decline reimbursement of THC-containing strains of cannabis (with or without CBD) in indications such as chronic pain, complex regional pain syndrome (CPS), and phantom pain. The agency admitted that the available evidence is limited and does not justify any form of copayment (i.e., reimbursement) by the Ministry of Health [10].

The regulations regarding treatment with cannabinoids are murky and split into numerous law acts; as a result, many physicians avoid prescribing cannabis, even when the treatment has already been prescribed by another qualified healthcare professional. Technically, every medical doctor in Poland is permitted to prescribe cannabis, regardless of having a specialist’s title or professional training, similarly to other medicines based on controlled substances, such as opioids [1,2,4,5,6]. On the other hand, the decision to initiate treatment and the responsibility for choosing proper indication and dosing relies solely on the prescribing physician because no official guidelines define these [11]. Patients receiving cannabinoids as therapy do not obtain official documents, such as certificates. No registry of patients taking cannabinoids exists to track prescriptions, overall consumption rates, or outcomes. There are also no official lists of physicians authorized to prescribe cannabis [1,2,4,5,6]. No independent source of education for physicians is available, and the medical uses of cannabinoids and the physiological basis of their mode of action are not part of medical curricula for medicine students. In the current setting, prescribing cannabis might be considered risky, especially given the lack of educational resources available to the HCPs. To date, no studies have been conducted in Poland to reveal the educational needs and preferences for systemic solutions to control the medical cannabis market.

Surveys regarding medical cannabis were conducted worldwide among certified medical doctors, pharmacists, and medical faculty students [12,13,14,15,16,17,18,19,20,21,22,23,24,25,26,27,28,29,30]. Most of these studies reveal problems with the lack of local clinical standards or knowledge about the legal status of medical cannabis [12,13]. In studies that included such questions, most respondents ranked their level of knowledge as low or insufficient in the context of clinical practice [14,15,16,17]. Furthermore, both students of medical faculties, including medicine, nursing, or pharmacy, and certified physicians declare they would like to receive more education. The students participating in these studies from other European countries (Spain, Poland, Serbia) also admit that they would like to have some classes at the university [19,20,21]. Previous studies demonstrate that, although most medical doctors accept using cannabis for medical reasons, recreational uses do not get as much support. Studies also show that views vary by medical specialty, gender, age, and religiosity [12,13]. Oncologists and palliative care specialists usually advocate strongly for the use of medical cannabis, family medicine, and neurology specialists are more conservative [12,13,22,23,24,25,26,27,28]. There are also slight differences in the acceptance of incorporating cannabinoids in clinical practice between doctors and medical students living in different geographical locations [12]. In both studies conducted among general practitioners in Ireland or Minnesota, 58% of physicians supported the legalization of cannabis for therapeutic purposes, and among Spanish nursing students—75% [18,19,23]. In Eastern Europe, this proportion was generally lower, reaching 33% in medical students of Belorussian origin. In Russia, only 26% of male students of medicine declared they would recommend medical cannabis to a patient in the event of its legalization [12]. These differences might be explained by personal experiences, professional training, and cultural differences. Previous studies have shown profound differences in illicit drugs and alcohol consumption among different European countries. There are also significant differences between the consumption of controlled substances in medicine between Eastern (less than 1000 defined daily dose (DDD) of morphine) and Western Europe (over 10,000 DDD) [29].

Medical cannabis remains an ongoing controversy in conservative countries of East-Central Europe; however, none of the studies mentioned above involved medical doctors from Poland. Poland has been a member of the United Nations (UN) since 1999, and of the European Union (EU) since 2004. Considering that the average age of medical doctors in Poland in 2017 was 52 years [30], and the time required to acquire specialist training, most attended university in the 80′. At that time, Poland was still under Soviet influence, which ended after 1989. Therefore, Polish citizens in recent decades have been under the influence of contrary cultures, which shaped their beliefs, perspectives, and actions. We decided to investigate their views on medical cannabis and legislative solutions and their self-evaluated knowledge level, educational needs, and motives for expanding their knowledge on medical applications of cannabinoids. This study aims to provide insights from medical practitioners to inform their views on the medical cannabis policy in Poland approximately three years after its legalization. We aim to use the results to inform relevant stakeholders about educational activities necessary to increase competence and knowledge and to motivate physicians to attend such events.

## 2. Materials and Methods

This study report was written based on the the Checklist for Reporting Results of Internet E-Survey (CHERRIES) guidelines [31]. It was an open survey, and participation was voluntary. Access to the questionnaire was only possible through a direct link. We offered no incentives for participation.

### 2.1. Survey Development

We initially aimed to perform the survey in traditional (paper) form; however, the form was switched to digital because of the coronavirus pandemic outbreak. We used Google Forms as a data collection platform. Two medical doctors from the research group tested the online survey before the links were shared with participants. They introduced minor corrections to the wording and mechanics of the survey, i.e., type of questions.

We based the survey on two self-developed questionnaires. One was previously distributed during a conference about medical cannabis in January 2019 organized by Hospice of St. Christopher in Warsaw. Questions regarding systemic solutions were adapted from that survey. These questions asked about the attitude towards medical cannabis legalization and formal restrictions and requirements for cannabis prescribing. The second survey was developed to investigate attitudes and knowledge about opioids among physicians, commissioned by the National Bureau for Drug Prevention (NBDP). Results from the survey about opioids were reported separately (the report is owned by NBDP and is not publicly available). We adapted and adjusted the questions regarding clinical aspects from that questionnaire on opioids, but we plan to report these results in a separate paper.

### 2.2. Survey Design

The study’s aims, information about the researchers’ organization, and a short survey description were displayed before the questions. The study questionnaire consisted of 57 items grouped in five parts: demographic data, legal aspects, and access to medication containing cannabinoids; cannabinoids’ use; clinical practice; educational needs; and personal experiences with controlled substances. There were open questions (about medical specialty or to allow participants to explain their answer in more detail) and closed questions (some using Likert 5-point scale or single- and multiple-choice answers). Questions were not randomized. The survey form forced its completion before submitting the result. All participants could check and correct their answers before submitting.

### 2.3. IRB Approval

The study protocol was prepared in line with the recommendations of the Helsinki Declaration. It was approved by the Bioethics Committee of the Medical University of Warsaw (IRB statement from the 3 February 2020, number AKBE/22/2020).

### 2.4. Participants

The survey participants were medical doctors with or without specialization. The participants were recruited from June to October 2020 in the following ways:

- From an online palliative medicine course in June and October 2020, which our research group organized in the Hospice of St. Christopher in Warsaw.
- From a closed Facebook group for young medical doctors during specialization (residents) known as “Residents Agreement”, with the group’s owner verifying the professional background of group members.
- Through a newsletter for medical doctors known as “Young Medical Professionals”, led by an organization which helps in the preparation of professional examination for prospective physicians.
- Through a personal network of physicians from different medical backgrounds.
- We sent a request to share the link to the questionnaire with all regional medical chambers in Poland. There are 16 chambers, one in each voivodeship. It is currently mandatory that each medical doctor is a member of one located in his primary workplace. Each has its website and a newsletter sent to every member.

The diversity of sources should enable the participation of a broad group of physicians representing different medical backgrounds from different age groups and geographical localization. In Poland, there are essential differences in terms of political preferences, religiosity between the eastern and western parts of Poland, with the east being more conservative and catholic and the west showing contrary tendencies. These factors influence choices and opinions about cannabis; therefore, it was vital to reach physicians using various channels.

We did not collect personal and contact data. To ensure the complete anonymity of the survey users, the IP was not collected. Each set of answers was reviewed manually for completeness and any random/accidental entries. None were identified.

We would exclude any entries made by physicians or double entries identified based on the open-ended answers. There were no exclusion criteria in terms of time spent on filling out the questionnaire. The survey forced the respondent to fill out all fields, and incomplete records were not saved.

### 2.5. Statistical Analysis

Statistical analysis was performed using the IBM SPSS Statistics ver. 26 (IBM Corp., Armonk, NY, USA). An analysis of basic descriptive statistics was performed.

## 3. Results

### 3.1. Response Rates

Due to the limitations of the online platform, which does not enable the removal of duplicates, it was necessary to review all records manually. Because some questions (e.g., medical specialty, years of practice, answers to some questions in the clinical part) had to be introduced by the survey participant in writing (e.g., medical specialties), it was possible to identify unique visitors. No duplicates were found.

### 3.2. Basic Characteristics

Between June and October 2020, 173 physicians from Poland completed the survey. Most participants (*n* = 150; 86.7%) were less than 50 years old and lived in large cities with more than 100,000 inhabitants (*n* = 112; 64.7%). Only a limited proportion declared they work mainly in the private sector (*n* = 36; 20.8%). The most common MDs with specialist titles were general practitioners (GPs), internal medicine, and oncology-related.

The participants were from 15 out of 16 voivodeships (macroregions) of Poland. A similar proportion of participants were recruited from western voivodeships in Poland (*n* = 66; 38.15%), central (*n* = 59; 34.1%), and eastern regions (*n* = 48; 28.78%). We presented other characteristics of the study participants in Table 1.

### 3.3. Education Level on Cannabinoids and Medical Cannabis

More than half of the study participants never received any education regarding medicinal cannabis (*n* = 104; 60.1%). The most mentioned educational activity was a lecture about cannabinoids attended at a conference dedicated to another medical subject (*n* = 49). Few participants participated in a conference (*n* = 7), or a course (*n* = 10) dedicated entirely to medical cannabis and cannabinoids before taking part in the study. When asked if their knowledge was sufficient to counsel patients on medical cannabis use, roughly 10% of doctors said they did (17/173) and 71.1% (123/173) said they did not. More details can be found in Table 2.

For the questions which used a 5-point Likert scale for assessment, we decided to group the answers to ease the interpretation. We ranked 4 and 5 as “agree”, 3 as “neutral opinion”, and 1 to 2 as “disagree”. In terms of the motivation for increasing knowledge on medical cannabis and cannabinoids, most frequently, the doctors claimed that they would like to be able to answer patient questions (160/173; 92.4%) and discuss their experiences with other medical professionals (159/173; 91.9%). Another reason frequently chosen was to seek new treatments for patients for whom other treatments do not provide sufficient relief or the side effects are intolerable (145/173; 83.8%). A similar number of participants declared having safety concerns (69/173; 39.9%) and denied having any (66/173; 38.2%). Personal motivation was the least motivating; only 38 participants agreed or partly agreed with that statement (22.0%). Only 38 participants (22%) declared that they were not interested in increasing their knowledge of medical cannabis. Table 3 shows the motives for further education on cannabinoids.

### 3.4. Systemic Solutions on Access to Medical Cannabis

Most study participants (161/173; 93.1%) declared a need to create clear guidelines for using cannabinoids in clinical practice. One hundred and twenty four participants (71.7%) believed that the Polish government should also reimburse medicines containing cannabinoids. Physicians showed less support for the herbal form of cannabis (90/173; 52.0%). Most doctors disagreed with the statement that an official request for cannabis treatment should be issued by a prescribing physician and approved by an officially established, empowered body before treatment initiation (142/173; 82.1%) or that, in all cases, the patient should be consulted by a psychiatrist before initiation of treatment with cannabinoids (108/173; 62.4%). The majority also disagreed that there should be a national registry of patients or doctors authorized to prescribe medical cannabis (90/173 in both cases; 52.0%). Fewer doctors disagreed (51/173; 29.5%) than agreed (85/173; 49%) that the prescription for cannabinoids should be issued only by a physician with training in cannabinoids use. It is worth noting that being a specialist was claimed necessary (72/173; 41.6%) and not necessary (79/173; 45.7%) by a similar proportion of physicians. We presented answers to the questions on systemic solutions in Table 4.

### 3.5. Recreational Drug Use

Questions about personal experiences with recreational drugs revealed that most of the participants were naïve about cannabis. Only 39.9% (*n* = 68) admitted having an experience with recreational cannabinoids, such as herbal cannabis and hashish. Most study participants (60.7%; *n* = 105) denied having any personal experience with cannabis. One of the participants claimed to be addicted to cannabis.

Those who admitted having used it consumed it for reasons other than medical (32.94%; *n* = 57). In addition, 82.1% (*n* = 142) declared that, if marijuana was legal, they would not use it for recreational purposes. Most agreed that cannabis can be addictive (90.2%; *n* = 156) and is harmful to human health (64.2%; *n* = 111); however. not with the statement that it might be a ‘gateway drug’ leading to abuse of hard drugs (59%; *n* = 102). Other results might are in Table 5.

## 4. Discussion

As the medical cannabis market grows in Poland, it becomes essential to understand physicians’ perspectives on this controversial topic. This study was the first performed among Polish physicians to investigate their beliefs about medical cannabis and views on the shape of policy in their country. Most participants (93.1%) confirmed a need to prepare medical guidelines about cannabinoids use. While most supported reimbursement of medicines containing cannabinoids (71.7%) for herbal cannabis, this percentage was lower (52%). We also found a legitimate need to provide educational interventions to train medical professionals in medical applications of cannabinoids and the physiological basis of their mode of action. Over 1/3 also declared that they have safety concerns regarding medical uses of cannabinoids (39.9%). It is worth noting that medicinal cannabis was also considered safer than one bought from illegal sources by the vast majority (97.7%); 71.1% claimed their knowledge level to be too low to provide advice to patients. Almost all physicians agreed (92.4%) that they would prefer to be more knowledgeable on this topic to fulfill patient’s expectations. The factors encouraging them to seek more information about medical cannabis and cannabinoids were mostly patient-centered (seeking new treatments for cases with no other acceptable therapeutic options, clarifying safety concerns). At the same time, most declared that personal motives are not a factor that could increase their willingness to learn more (only 22% agreed).

A lack of clear guidelines regarding medical applications of cannabinoids is one of the commonly listed barriers to cannabis use [12,16,22]; 93.1% of Polish physicians declared a need to formulate clear guidelines to inform physicians how to incorporate cannabinoids into their clinical practice. Similar findings come from studies conducted in other countries; 93% of prospective medical doctors from Serbia and 64% of physicians from Canada also claimed that such guidelines are essential [12,16]. Still, a survey covering European countries demonstrated that medical associations published position papers about medical cannabis and cannabinoids in only three of them [11]. Given that cannabis use and patient demand are increasing, the unresolved issue of formulating clinical management guidelines should be addressed urgently by relevant medical associations in Poland.

Over 71% of physicians declared that their knowledge level about cannabinoids is “not” or “rather not” sufficient for patient counseling. Our finding that MDs self-evaluated knowledge about cannabinoids is not sufficient is repeatedly found in studies from other countries [12,13,14,15,16,17,18,19,20,21,22,23,24,25,26,27,28]. In Norway, 71% of surveyed physicians declared that they would like to be more knowledgeable about this topic [22]. Similarly, 74% of Canadian physicians felt not sufficiently informed about medical cannabis [24]. In another study conducted among physicians from Minnesota, 50% felt prepared for answering patient questions, and 77% were interested in learning more, in comparison with 92% of participants of our study [23]. In another study with a mixed group of HCPs (including pharmacists and nurses) involved in oncology care, the percentage of professionals who self-evaluated their knowledge level to be too low was 84%, similar to our findings [28].

Studies conducted among healthcare practitioners (including prospective HCPs) show that, even when the proportion of participants with enough confidence to counsel patients is relatively low, the support for the use of cannabinoids remains high for patients with short life expectancy or no alternatives for treatment [12,13,18,22]. This group encompasses terminal cancer patients and patients with symptoms refractory to standard treatment, such as cancer and neuropathic pain. In most studies among physicians, pain and cancer-related symptoms are most commonly mentioned as indications for cannabis use [12,13]. In an Irish study, 63.5% of physicians claimed that cannabinoids might be prescribed for pain management, and 68%-in terminal patients [18]. Additionally, 88% of physicians from Norway chose adverse effects of cancer treatment as an indication [22]. Similar findings described Gardiner et al. in their systematic review among oncologists and general practitioners [13].

Solid background education increases the confidence in using cannabinoids in clinical practice. Studies were also conducted among physicians in Europe, includng those with either specialist training or those who received training in the treatment of addictions were more inclined towards using medical cannabis [12,18,22]. Furthermore, 70% of physicians from Canada indicated that they would be more comfortable if they had received any formal education [13]. Another systematic literature review of studies conducted among representatives of medical professions has demonstrated a lack of knowledge as a barrier for authorizing treatment with cannabis [13]. Results from these studies underline the importance of educational interventions that would explain basic scientific concepts and give practical instructions on how to use medical cannabis.

The lack of professional training declared by Polish physicians is concerning. Previous studies have found that the source of information consulted for healthcare professionals’ clinical practice is also questionable. Previous studies have shown that their primary source of knowledge was seldom medical courses or medical faculty, but rather media and the internet [12,13]. In a Norwegian study, news and television were the most frequently mentioned sources of knowledge, indicated by 39% of physicians, whereas medical literature was picked by roughly a quarter [22]. Likewise, a survey among Canadian hospital pharmacists demonstrated that most (66%) did not receive any formal education about cannabinoids. They also admitted that the sole educational resource available is an online self-study course [13]. News or websites dedicated to medical cannabis often raise unrealistic expectations about its properties and propagate information only partly based on medical literature. Furthermore, in reliable, evidence-based websites for Polish physicians, medical use of cannabinoids topic is somewhat absent. Access to professional training is also problematic for young adepts of medical sciences. Currently, medical curricula at Polish universities do not include the endocannabinoid system and pharmacology of cannabinoids. This problem could be solved by incorporating the pharmacological aspects (including the ECS physiological and pathophysiological aspects) and clinical use as a part of pain treatment courses, free conferences run by regional medical chambers, and other independent sources of training for certified HCPs, and also by introducing these topics to curricula at Polish medical universities.

Our results show that there is a need for such educational interventions for Polish medical doctors. As indicated in previous studies, professional training encourages the use of cannabinoids. It is essential for patients for whom it is impossible to offer alternative treatments or available methods that were not well-tolerated. Proper training also helps medical professionals identify and advise against their use in patients for whom cannabinoids might be harmful. However, it is worth noting that a self-assessed level of knowledge, often used in such studies, should not be perceived as equal to a measure of competence. Previous research in various domains has shown that doctors tend to underestimate their actual level of knowledge or overestimate it, regardless of training and specialty and the domain of self-assessment [32]. Similar findings were the outcome of studies among other professions [33]. Therefore, self-evaluation is instead a measure of confidence than a factual knowledge level. A study by Zolotov et al. revealed that doctors who were more likely to recommend medical cannabis had less confidence in their knowledge about medical cannabis [15]. We agree with the authors that this could mean that individuals with higher awareness are more knowledgeable of the uncertainties regarding the medical uses of cannabinoids. In summary, these aspects should be considered when interpreting the results of our study.

In one of the previously conducted studies among medical students in Russia, religiousness significantly correlated with a more restrained approach to using cannabinoids in medicine; 57% of secular students declared they would recommend cannabis to a patient, in comparison with 27% of religious students (*p* < 0.001), and more often claimed it has positive effects on physical and mental health (54.3% vs. 28.2%; *p* < 0.01) [12]. Poland is considered a conservative country, with almost 89% of citizens declaring themselves as catholic, according to the Chief Statistical Office [34]. Interestingly, most physicians participating in the survey opted for solutions giving the most freedom when deciding how to approach a potential medical cannabis patient. Any restriction in prescribing, such as the requirement of having a specialist title, approval of regulatory body for medical cannabis treatment, or a formal requirement of obtaining a second opinion of a psychiatrist, was considered redundant. However, it is worth noting that they might have been taking into account other factors, such as long waiting times for additional consults, the cost for the patient, others which would cause a delay in initiating treatment. and, as a result, the alleviation of the patient’s ailments.

Nonetheless, in other European countries, such as the United Kingdom and Ireland, having a specialist title is one of the mandatory requirements for the prescription of cannabis [11,35]. The European Pain Federation (EFIC) report indicates that this state of affairs opposes trends found in other European countries. For the most part, medical cannabis is available to well-defined patient populations, and how it is dispensed is tightly controlled [11]. In several other countries, according to a report published by the European Pain Federation, access to medicinal cannabis is limited in other ways. For example, it is only available through compassionate use programs (in Sweden, Norway), or, although technically legal, the supply of cannabis for medicinal purposes is not available (in Slovenia) [11]. Poland, in contrast, presents a liberal approach to medical cannabis in the current legal framework, where there is no approved dosing indications list. The MoH requires no additional training or certificates for prescribing herbal cannabis. Therefore, the clinical decision to initiate treatment in each case and dosing or duration of treatment rely solely on the prescribing physician [36].

Contrary to prescribing controlled substances, such as cannabinoids in Poland, the restrictions are tighter in other medical fields. At times, the reasoning behind those regulations is difficult to understand. Before the COVID-19 pandemic, according to the act on preventing and combating infections and infectious diseases in Poland, performing qualification for vaccination and the procedure itself was only possible for healthcare professionals who had attended an additional certified vaccination course or received this training during the specialization [37]. Although vaccine hesitancy is primarily based on the uncertainties regarding the safety profile of vaccines, the risk-benefit profile of vaccination in both the short- and long-term favors its use indisputably [38,39]. Vaccination is a very safe procedure, and no long-term side effects that would be unquestionably related to vaccination were identified [39]. Paradoxically, with current regulations, it is much easier for patients to buy opioids and cannabinoids, who now require only one visit to the doctor’s office and pharmacy before getting vaccinated. To receive a vaccine, at least two visits to the doctor’s office are necessary (one to get a prescription and then the other to get qualified for vaccination), and 1–2 visits at the pharmacy, depending on the availability of the vaccine.

Prescribing controlled substances is associated with the risk of developing dependence and abuse [40]. Additionally, in the case of opioids, abuse might result in fatal overdose in the sole 2019 in the USA, whereby more than 14,000 cases of deaths related to consumption of prescription opioids were recorded [40,41]. Even though the risk associated with medical uses of cannabinoids is incomparable to opioids, improper use can lead to addiction, traffic accidents, and risks to children who accidentally consume it [42,43,44]. Therefore, an optimal solution would embrace much tighter regulation of medical cannabis prescribing. In 2020, electronic prescriptions were introduced and made mandatory in Poland and replaced the paper form. This system allows physicians and patients to track prescriptions made in the past easily. It also requires the physician to confirm his identity before issuing any prescription via an official certificate or bank account [45]. Such a system could also enable rapid assessment of the situation and catch worrying phenomena related to prescription narcotic substances early on. In New York, mandatory electronic prescribing of controlled substances (including opioids) has reduced the number of prescriptions by 53% [46]. In Poland, to date, the consumption of medications using controlled substances is low (<2000 DDD for opioids), but there are some increasing trends observed for some of these substances, such as tramadol, which needs further monitoring [29]. Therefore, some control over prescriptions involving controlled substances should be warranted, which might be implemented as part of the electronic prescriptions system.

This study has some limitations. The most important is the small study size which does not enable a subgroup analysis to confront opinions of medical professionals of different backgrounds and identify associations of different demographic factors with participant’s decisions. A significant limitation is that we have only targeted physicians and not representatives of other medical professions such as nurses and pharmacists, who are also directly involved in patient care. Because it was an open survey, people with more interest in medical cannabis were more likely to participate, and, as a result, we might not have recruited a representative sample. The proportion of MDs with experience with recreational cannabis (39.9%) was higher than according to a survey conducted among the general Polish population aged 15–64 years (7.7% for females and 16.4% for males) [47]. It could have influenced the acceptance of cannabinoids as medicines and support for their legalization. However, considering that the participation was voluntary and anonymous, and that no tracking data were collected, the answers were more sincere than in the study conducted by National Bureau for Drug Prevention.

## 5. Conclusions

This study is the first insight into the perspectives of Polish physicians regarding the medical applications of cannabinoids. Most medical professionals have expressed their support for the medical use of cannabis and cannabinoids. However, the majority do not feel prepared for patient counseling, which is concerning. They could benefit from targeted educational interventions. Such interventions might encompass independent courses for licensed physicians, congresses, and websites. Additionally, this study has also identified physicians’ preferences and suggestions for stakeholders responsible for shaping the policy regarding cannabis-based therapeutics. Further research is encouraged to investigate opinions and knowledge of the representatives of other medical professions (such as pharmacists, nurses, among others); to detect differences among representatives of different medical specialties, such as general practitioners, oncology, and pain specialists; and to identify factors contributing to their choices.

## Figures and Tables

**Table 1:** Demographic data.

Age Group	<30 Years	41	23.7
	30–39 years	76	43.9
	40–49 years	33	19.1
	50–65 years	23	13.3
Gender	Male	59	34.1
	Female	114	65.9
Medical background	Internal medicine (and associated specialties)	38	
	GP	20	
	Oncology and hematology (e.g., radiotherapy. oncology surgery)	14	
	Psychiatry	11	
	Anesthesiology and intensive therapy	16	
	Neurology (adult and pediatric)	7	
	Gynecology	5	
	Surgical	6	
	Palliative care	3	
	Hematology	2	
	None/during medical training (unspecified)	28	
	Other	24	
	pediatrics	7	
	Medical internship	5	
Primary workplace	Town/villages up to 10,000 habitants	12	9
	Towns from 10–20,000 habitants	15	8.7
	Cities 20–50,000 habitants	9	5.2
	Larger cities 50–100,000 habitants	25	14.5
	Large cities	112	64.7
Primary sector	Public	136	78.6
	Private	36	20.8
	No data	1	0.6
Contact with persons with addictions	Yes	76	43.9
	No	97	56.1
Past use of recreational cannabis	Yes	68	39.9
	No	105	60.7
Clinical experience with cannabinoids	Yes	15	8.7
	No	158	91.3

GP—general practitioner.

**Table 2:** Education on medical cannabis and cannabinoids.

	Answer	N (%)
Did you receive any education on medical cannabis and cannabinoids in the past?	No	104 (60.1%)
	Yes	69 (39.9%)
What kind of education was it? *	A conference dedicated to cannabis/cannabinoids	7
	Course on medical cannabis and cannabinoids	10
	A lecture on cannabinoids during another conference	49
	Other	22
Do you believe your knowledge is sufficient to counsel patients on cannabinoid use?	No	80 (46.2%)
	Rather not	43 (24.9%)
	Neither agree nor disagree	33 (19.1%)
	Rather yes	12 (6.9%)
	Yes	5 (2.9%)

* the number exceeds the number of participants declaring past training in cannabinoids as some marked more than one answer.

**Table 3:** Motivation for expanding knowledge on medical uses of cannabinoids.

	1— Disagree	2—Partly Disagree	3— Neither Agree Nor Disagree	4— Partly Agree	5— Agree	Mean	Median	Mode
I’m not interested in medical uses of cannabinoids	110	25	13	12	13	1.8	1	1
I would like to expand my knowledge and skills in the medical use of cannabis/cannabinoids	3	5	17	47	101	4.38	5	5
I would like to be able to answer patient questions about cannabinoids	4	3	6	49	111	4.5	5	5
I would like to be able to consult cases from my clinical practice with other professionals	3	3	8	53	106	4.48	5	5
I would like to be able to verify my experiences and opinions related to cannabinoid containing products	3	4	11	59	96	4.39	5	5
I have some concerns about the safety of cannabinoids	30	36	38	54	15	2.93	3	4
Seeking new treatments for patients for whom current treatments are ineffective or intolerable	6	5	17	49	96	4.29	5	5
Personal motivation	54	29	52	23	15	2.51	3	1

SD—standard deviation.

**Table 4:** Access to cannabinoid, systemic, medicine-related solutions.

Do You Agree That	1—Disagree	2—Partly Disagree	3—Neither Agree Nor Disagree	4—Partly Agree	5—Agree	Mean	SD (±)	Median	Mode
any physician may prescribe medical cannabis and cannabinoids?	32	26	31	35	49	3.25	1.48	3	5
medical cannabis and medicines containing cannabinoids should be available to patients in selected indications only with a physician’s prescription with a specialist background?	48	31	22	28	44	2.94	1.57	3	1
medical cannabis and medicines containing cannabinoids should be available with a doctor’s prescription with special training (or certificate) in treating cannabinoids?	31	20	37	38	47	3.29	1.44	3	5
A request for cannabinoid treatment issued by a physician should be approved by a government or self-regulatory medical organization	109	33	17	6	8	1.68	1.09	1	1
initiation of cannabinoid treatment, regardless of the primary indication, should be consulted with a psychiatrist	55	53	30	21	14	2.34	1.26	2	1
there is a need for clear guidelines for medical cannabis/cannabinoid drug treatment	1	2	9	28	133	4.68	0.68	5	5
medicines containing cannabinoids should be reimbursed	4	3	42	44	80	4.12	0.99	4	5
herbal cannabis should be reimbursed	11	13	59	32	58	3.65	1.2	4	3
there should be a national registry of people treated with cannabinoids	64	26	37	21	25	2.52	1.45	2	1
there should be a national registry of physicians authorized to prescribe cannabinoids	63	27	33	21	29	2.57	1.49	2	1

SD—standard deviation.

**Table 5:** Past use of cannabis and other psychoactive substances.

	No	Yes	All
Have you used cannabinoids (marijuana, hashish) in the past?	105 (60.7%)	68 (39.9%)	173 (100%)
If yes, have you used cannabinoids for medicinal purposes?	57 (32.94%)	11 (6.36%)	68 (39.3%)
Do you think cannabinoids (marijuana, hashish) are dangerous to human health?	93 (53.8%)	80 (46.2%)	173 (100%)
Do you consider yourself to be addicted to cannabinoids?	172 (99.4%)	1 (0.6%)	173 (100%)
Do you believe that cannabinoids (marijuana, hashish) can be addictive?	17 (9.8%)	156 (90.2%)	173 (100%)
Do you think that the use of cannabinoids (marijuana, hashish) leads to the abuse of harder drugs?	102 (59.0%)	71 (41.0%)	173 (100%)
Do you believe that marijuana/hashish is harmful to human health?	62 (35.8%)	111 (64.2%)	173 (100%)
Do you think medical marijuana is safer than illegal cannabis products (“street cannabis”)?	4 (2.3%)	169 (97.7%)	173 (100%)
Is it possible that when you have easy access to medical marijuana, you will reach for it for recreational purposes?	142 (82.1%)	31 (17.9%)	173 (100%)
Have you used other psychoactive drugs in the past (e.g., opioids, LSD, ecstasy, psilocybin, legal highs), or/are you currently doing so?	109 (63.0%)	21 (12.1%)	130 (75.1%)

## Data Availability

Source data is available from the corresponding author upon a reasonable request.
